# Indoor Particulate Matter From Smoker Homes Induces Bacterial Growth, Biofilm Formation, and Impairs Airway Antimicrobial Activity. A Pilot Study

**DOI:** 10.3389/fpubh.2019.00418

**Published:** 2020-01-24

**Authors:** Emma M. Stapleton, Robert Manges, Gavin Parker, Elizabeth A. Stone, Thomas M. Peters, Robert J. Blount, Julio Noriega, Xiaopeng Li, Joseph Zabner, Philip M. Polgreen, Octav Chipara, Ted Herman, Alejandro P. Comellas

**Affiliations:** ^1^Department of Internal Medicine, Roy J. and Lucille A. Carver College of Medicine, University of Iowa, Iowa City, IA, United States; ^2^Department of Chemistry, College of Liberal Arts and Sciences, University of Iowa, Iowa City, IA, United States; ^3^Department of Occupational and Environmental Health, College of Liberal Arts and Sciences, University of Iowa, Iowa City, IA, United States; ^4^Department of Computer Science, College of Liberal Arts and Sciences, University of Iowa, Iowa City, IA, United States

**Keywords:** particulate matter, indoor environment, COPD - chronic obstructive pulmonary disease, airway surface liquid (ASL), innate host defense

## Abstract

**Background:** Particulate matter (PM) air pollution causes deleterious health effects; however, less is known about health effects of indoor air particulate matter (IAP).

**Objective:** To understand whether IAP influences distinct mechanisms in the development of respiratory tract infections, including bacterial growth, biofilm formation, and innate immunity. Additionally, we tested whether IAP from Iowa houses of subjects with and without recent respiratory exacerbations recapitulated the National Institute of Standards and Technology (NIST) IAP findings.

**Methods:** To test the effect of NIST and Iowa IAP on bacterial growth and biofilm formation, we assessed *Staphylococcus aureus* growth and *Pseudomonas aeruginosa* biofilm formation with and without the presence of IAP. To assess the effect of IAP on innate immunity, we exposed primary human airway surface liquid (ASL) to NIST, and Iowa IAP. Lastly, we tested whether specific metals may be responsible for effects on airway innate immunity.

**Results:** NIST and Iowa IAP significantly enhanced bacterial growth and biofilm formation. NIST IAP (whole particle and the soluble portion) impaired ASL antimicrobial activity. IAP from one Iowa home significantly impaired ASL antimicrobial activity (*p* < 0.05), and five other homes demonstrated a trend (*p* ≤ 0.18) of impaired ASL antimicrobial activity. IAP from homes of subjects with a recent history of respiratory exacerbation tended (*p* = 0.09) to impair ASL antimicrobial activity more than IAP from homes of those without a history respiratory exacerbation. Aluminum and Magnesium impaired ASL antimicrobial activity, while copper was bactericidal. Combining metals varied their effect on ASL antimicrobial activity.

**Conclusions:** NIST IAP and Iowa IAP enhanced bacterial growth and biofilm formation. ASL antimicrobial activity was impaired by NIST IAP, and Iowa house IAP from subjects with recent respiratory exacerbation tended to impair ASL antimicrobial activity. Individual metals may explain impaired ASL antimicrobial activity; however, antimicrobial activity in the presence of multiple metals warrants further study.

## Introduction

Health impacts of acute ambient particulate matter (PM) exposure, especially fine (≤ 2.5 μm) PM, are well-documented and include an increased risk of respiratory infections, especially in susceptible populations (1–7).

Airway innate immune mechanisms, such as airway surface liquid (ASL) antimicrobial activity, play an important role in the development of respiratory infections, as well as the mechanism of chronic lung diseases such as cystic fibrosis (8, 9). The ASL is a thin layer of liquid coating the lungs and is responsible for immediately killing incoming pathogens. It is composed of various antimicrobial peptides and proteins (AMPs), such as β-defensins, lactoferrin, lysozyme, and surfactant proteins, among others, and is instrumental in airway innate immunity (10–13).

Air contaminants, such as PM, can impair airway innate immunity and reduce immediate ASL bacterial killing (14, 15). Additionally, transition metals in PM, including iron, can affect airway innate immunity, which can lead to enhanced bacterial growth and biofilm formation (16). Our laboratory has studied the effect of outdoor ambient PM on ASL antimicrobial activity as well as its effect on bacterial growth and biofilm formation (15, 17).

Additionally, a recent area of interest in the environmental science community is the effect of indoor PM on lung health, especially since we spend the majority of our time in buildings (18, 19). Chronic exposure to indoor air pollution could have a disproportionately high impact on overall daily PM exposure. Indoor air quality can be affected by outdoor pollutants entering the home, as well as from indoor sources such as building materials, cleaning and cooking products, or combustion bi-products (20). However, there is a paucity of data regarding how indoor PM may influence mechanisms of airway innate defense mechanisms, despite the disproportionate amount of time we spend indoors.

To this end, we hypothesize that National Institute of Standards and Technology (NIST) standard indoor air particulate matter (IAP) will enhance bacterial growth and biofilm formation (*Staphylococcus aureus, Pseudomonas aeruginosa*), and impair bacterial clearance in ASL from airway epithelial cells (AECs). Additionally, we hypothesize that these results will translate using IAP collected from Iowa homes. To test this, we carried out a pilot study in 21 homes of current and former smokers, with and without a history of respiratory exacerbations, to assess whether IAP collected from their homes would affect *in vitro* bacterial growth, biofilm formation, and ASL antimicrobial activity.

## Methods

### IAP's Effect on *S. aureus* Growth in Minimal Media

To test if the NIST Standard Reference Material^®^ 2584 IAP enhances bacterial growth, we suspended 190 μL of log-phase bioluminescent *S. aureus* (Xen29, Caliper Lifesciences Bioware^®^) isolated from a human strain and modified [single-copy of *Photorhabdus luminescens* luxABCDE operon on the chromosome (17)], in a 10 mM NaPO_4_ buffer with 1% TSB in a 96-well plate (OptiPlate™, PerkinElmer, USA) maintained at 37°C, for details see Supplementary Material. We used two concentrations of the IAP (10 and 50 μg/mL), suspended in minimal media, which were plated aside vehicle control. Relative light units (RLUs) were read hourly for 3 h. Hourly growth was quantified as percent growth relative to RLUs at time 0, per condition. Upon termination of the bacterial growth assay, bacteria were aspirated and killed with bleach in a biosafety compliant manner. Any disposable materials used in the assay were sanitized with bleach and disposed of in a biosafety container.

### IAP's Effect on *P. aeruginosa* Biofilm Formation

We used *P. aeruginosa* [PA01, (21)] that expresses the pMRP9-1 plasmid constitutively to test for biofilm formation, adapting methods previously described (22–24). We cultured *P. aeruginosa* (PA01, OD_i600_ = 0.01) using MBEC^TM^ Calgary (Innovotech, Edmonton, Alberta, CA) 96-well biofilm inoculator plates in M63 minimal media supplemented with 0.4% arginine for 24 h, then exposed the inoculum to challenge conditions containing doses of 10 or 50 μg/mL of NIST IAP (*n* = 12). We then stained inoculated pegs with 0.1% crystal violet and eluted them in 30% glacial acetic acid. We read the OD (550 nm) for each sample and compared to the control condition (*P. aeruginosa* suspended in UltraPure^TM^ distilled water, Life Technologies^TM^) to quantify percent biofilm formation. Upon termination of the biofilm assay, bacteria were aspirated and killed with bleach and disposed of in a biosafety compliant manner. Any disposable materials used in the assay were sanitized with bleach and disposed of in a biosafety container.

### ASL Antimicrobial Activity

ASL was collected from primary human airway epithelium (HAE) cell cultures, from the University of Iowa cell culture core, grown at the air–liquid interface, as previously described (with modifications) (25). Briefly, we serially washed cell apical surfaces (four wells per donor) three times with 120 μL of 10 mM NaPO_4_ buffer (Ca and Mg free) every two days, and froze the samples at −80°C. Cell media (USG with antibiotics) was changed every 3 days to maintain cell viability (25).

We tested immediate bacterial killing, as defined by percent live bacteria within 20 min. We provide the specific timepoint analyzed for each of the experiments. Ten microliter of bacteria, suspended in minimal media (10 mM NaPO_4_, TSB concentration_Final_ = 1%), was injected into a 10 μL solution of ASL combined with 1 μL of the particle solution, field blank (FB) solution, or water. The 96-well plate was maintained at 37°C and bacterial luminescence, quantified as RLUs, was read at a 527 nm wavelength after 16 min.

For NIST IAP, we calculated the percentage of live *S. aureus* bacteria remaining (within 10 min) to the RLUs at *t* = 0 for each condition. For the IAP from Iowa homes, the percentage of live bacteria remaining was calculated by comparing ASL in the presence of each sample to the ASL treated with the FB control. ASL controls (12 from three donors) with 1 μL H_2_O were interspersed throughout the plate to account for any potential evaporation. Bacterial killing, as measured by RLUs, has been previously validated using colony forming units (17).

To test the effect of the soluble portion of the particles to inhibit bacterial killing, we used the previous method. However, before applying the particle mixture to the ASL, we centrifuged the particles (RPM = 12,000) for 4 min, then added the soluble portion of the particle mixture to the ASL and compared the percent of live bacteria within 10 min to live bacteria at the initial reading (0 min), as the soluble portion did not have physical particles to interfere with RLUs.

### Study Population

We recruited subjects from an NIH funded cohort (COPDGene, http://www.copdgene.org/) who reside in Iowa. All methods were performed in accordance with the relevant guidelines and regulations, informed consent was obtained, and no human tissue samples were used. Subjects were selected based on respiratory exacerbation history and their house location (within a 30-mile radius of the University of Iowa Hospital). Our study radius facilitated study adherence with elderly subjects and allowed for timely device transfer/maintenance. Selection aims were: similar GOLD (Global Initiative for Obstructive Lung Disease Criteria for COPD) stage and 50% male/female ratio. We recruited a total of 13 female and 8 male subjects who are current (*n* = 2) and former smokers (*n* = 19), with and without respiratory exacerbations based on history prior to enrollment (within the previous 3 years-−2015–2017). To be considered within the exacerbator group, participants were required to experience ≥1 exacerbation per year within the previous 3 years. Non-exacerbators had not experienced any exacerbations within the same time-frame. These subjects were followed from November 2016 to April 2017. Participants were provided with a survey (Supplemental Table 1) which inquired about potential IAP generating sources, and personal habits that may affect exposures, such as cleaning methods, cooking materials and combustion sources (20).

### Aerosol Collection

Particles were collected over 1 month during winter, to reduce infiltration from ambient particles and isolate particles generated in the home. We collected particles ≥20 nm using electrostatic precipitator (ESP) devices (OION B-1000, OION Technologies). We requested that the ESP be placed in the room most frequently occupied by study participants, typically the living room. ESP particle collection efficiency using our method varies depending on the type and size of a particle, with water soluble particles preferentially recovered; however, the OION ESP particle collection efficiency for a typical outdoor contaminant (Arizona road dust) is 65% (26).

The ESP generated 0.036 mg/min of ozone, and at a ventilation rate of 230 L/min the ESP would not exceed NAAQS exposure limits. All homes participating had active ventilation systems in place (26).

### Removing Particles From ESP and Filters

ESP collection plates are flat, which enables easy PM recovery. Twenty-one ESPs were wiped with eight wet (DI H_2_O) PVC filters (26). These filters were allowed to dry at least 72 h, then weighed and chemically analyzed using a microwave digestion methodology, with modified parameters to increase the recovery of metal analytes (27, 28). FBs were treated identically, where a wet PVC filter was used to wipe a clean ESP. The filter with a mass closest to 5 mg was submerged in a 5 mL conical tube. In one home, four of the five filters masses were >15 mg, in which case the filter with the lowest of the four masses was chosen. We used 1 mg/mL (mass/water) to normalize for particle dose. The mean volume of water used in all samples was added to the FB filter (3.7 mL).

To recover the soluble portion of PM, preferentially selected in our PM collection method, we cut PVC filters into strips, and submerged in distilled water (UltraPure™ Distilled Water, Invitrogen, Life Technologies, Grand Island, NY, USA). Conical tubes containing the 22 filters (21 homes, one FB) and H_2_O were placed overnight (18 h) on a titer-plate shaker (Lab-line instruments, Inc. Melrose Park, IL, USA) at a setting of 6 (~750 RPM) at room temperature. The following morning, the soluble, “supernatant” portion of the remaining suspension was isolated and frozen. Any remaining solution was also frozen at −4°C. Although the indoor PM samples represent only the soluble component of the IAP, we hereafter refer to it as IAP for consistency.

### Bacterial Colonization of Filter

The soluble portion of the IAP was tested for biological organisms by Matrix Assisted Laser Desorption/Ionization-Time of Flight Mass Spectrometry, as previously described (29). Briefly, the sample was mixed uniformly in a large matrix, which then absorbs ultraviolet light (nitrogen laser light, wavelength 337 nm) and converts it to heat energy. A small portion heats in nanoseconds and is vaporized with the sample. Time of flight differs according to the value of ionic mass-to-charge ratio. This value is compared to a strain-identification database.

### Participant IAPs' Effect on Bacterial Growth, Biofilm Formation

To assess whether IAP from homes of Iowa smokers influenced *S. aureus* growth, we used the previously described method; however, added only the soluble portion of IAP from homes to the bacterial suspension at a dose_initial_ of 50 μg/mL.

To test the effect of Iowa smokers' IAP on *P. aeruginosa* biofilm formation, we exposed inoculator lids to challenge conditions containing each indoor PM sample for 24 h. Following the particle challenge, inoculated pegs were stained using 0.1% crystal violet and eluted in 30% glacial acetic acid. OD_550_ was read for each sample as a measure of biofilm formation.

### Effects of Metals on ASL Antimicrobial Activity

The two most abundant metals, by mass fraction, present in the Iowa samples (aluminum and magnesium) were selected and analyzed for their effect on immediate ASL antimicrobial activity (30). We then combined AlCl_3_ and MgCl_2_ (>98% purity, Fischer Scientific, Pittsburgh, PA, USA) in an aqueous solution using DI water, to match their average IAP proportion and analyzed their effect on ASL antimicrobial activity. Furthermore, we investigated the effect of copper on antimicrobial killing. Because Cu is a divalent cation, it also has the potential to inhibit ASL killing; however, Cu is also a known antimicrobial agent, therefore its effect on ASL bacterial killing was of interest. We tested CuCl_2_ (>98% purity, Fischer Scientific, Pittsburgh, PA, USA) with human large airway ASL and *S. aureus*. Lastly, individual metal mass fractions were compared to bacterial killing in the presence of IAP.

### Statistical Analysis

We used Graph Pad Prism software, version 8.2.0. Significance was determined at α ≤ 0.05. To compare bacterial growth for each condition, we used unpaired two-tailed *t*-tests comparing growth under each condition to the control (*n* = 4 per condition). We tested for differences in biofilm growth from vehicle control (OD_550_) using unpaired two-tailed *t*-tests. For Iowa household IAP, bacterial growth at 4 h using a field-blank control was compared to each condition using Brown-Forsythe and Welch ANOVA tests, and biofilm formation was assessed in the same manner.

To analyze significance between immediate bacterial killing per condition we compared the percent of live *S. aureus* remaining within 10 min in the presence of ASL with and without exposure to whole-particle NIST IAP (50 μg/mL dose), compared to percent live bacteria in vehicle (SPB) with particle control at the same time-point. ASL control was compared to ASL+IAP with a paired two-tailed *t*-test. To test for differences between the percent of live bacteria between the control and soluble portion of the particle mixtures, live bacteria within 10 min was compared to *t* = 0 min for ASL alone, and live bacteria within 10 min in the presence of the soluble portion of NIST IAP (50 μg/mL dose_initial_) was compared to *t* = 0 min at the same condition. Paired, two-tailed *t*-tests were used to compare the percent of live bacteria within 10 min to live bacteria at the initial reading (0 min), as the soluble portion did not have physical particles to interfere with RLUs.

Bacterial growth and biofilm formation were assessed using a Brown-Forsythe and Welch ANOVA test where bacterial growth and biofilm formation from each home was compared to their respective vehicle control. To test for differences between each house's IAP and ASL with the field-blank (filter) control, live bacteria at 16 min per condition was compared to initial RLUs (0 min.) and converted to a percentage of live bacteria. The percentage of live bacteria remaining after ASL was treated with each participants' IAP was compared to the ASL control's (percentage of live bacteria) using paired *t*-tests. Bacterial killing in the presence of metals was calculated the same way, but live bacteria at 6 min was per condition was compared to initial RLUs (0 min.), using paired *t*-tests to compare untreated ASL to each dose of each metal. ASL antimicrobial activity between groups (exacerbators vs. non-exacerbators) was assessed using unpaired *t*-tests.

To test whether Global Obstructive Lung Disease (GOLD) status modifies the effect of percent live bacteria on exacerbation status we fit a logistic regression model with an interaction term for GOLD status and percent live bacteria using Stata/SE (v15.1, StataCorp., LLC, College Station, Texas, USA). Briefly, a “low” GOLD score was defined as GOLD zero (*n* = 13), and a “high” score was defined as GOLD one or two (*n* = 7). The GOLD status for one participant was not available, and this participant was therefore not included in the aforementioned analysis.

## Results

### IAP's Effect on Bacterial Growth and Biofilm Formation

**Figure 1:**
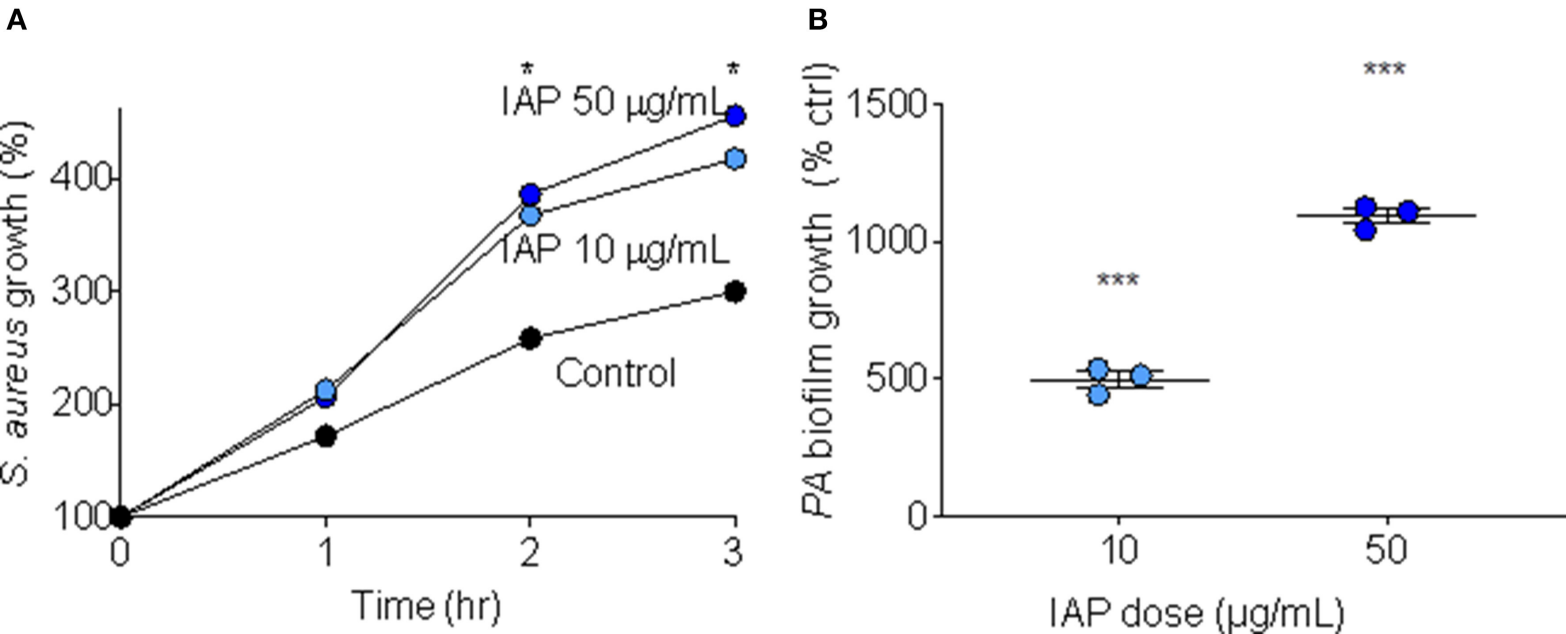
Bacterial growth and biofilm formation in presence of NIST IAP. **(A)** IAP enhances *S. aureus* bacterial growth in minimal media after 2 and 3 h, unpaired twotailed *t*-tests compared growth per condition to control (*n* = 4 per condition), ^*^p < 0.05 **(B)** *P. aeruginosa* biofilm growth in the presence of IAP particles at two doses (10 and 50 μg/mL). Error bars represent means and standard error of the means. Differences in OD_550_ of biofilm growth compared to vehicle control were compared using an unpaired two-tailed *t*-test ^***^p < 0.001.

In order to test whether IAP influences bacterial growth, we added two doses (10 and 50 μg/mL) of NIST IAP to *S. aureus* suspended in minimal media (10 mM NaPO_4_ buffer with 1% TSB), and assessed bacterial growth over time (RLUs at one, 2 and 3 h compared to RLUs at inoculation). After 2 and 3 h, NIST IAP significantly enhanced *S. aureus* growth (*p* = 0.03, 0.02; *p* = 0.04, 0.02, respective to dose and time) relative to bacteria without particles, Figure 1A, recapitulating previous observations of *S. aureus* growth in the presence of the same dose of an ambient pollutant [50 μg/mL, Coal Fly Ash (CFA)] (17). We used *P. aeruginosa* to assess whether IAP influenced biofilm growth by culturing *P. aeruginosa* in minimal media (M63 + 0.4% arginine) for 24 h, then exposing the inoculum to 10 or 50 μg/mL NIST IAP. We compared OD for each sample with *P. aeruginosa* suspended in vehicle control. As seen in Figure 1B, the percent of biofilm formation was increased using NIST IAP in a dose-dependent manner.

### ASL Antimicrobial Activity

**Figure 2:**
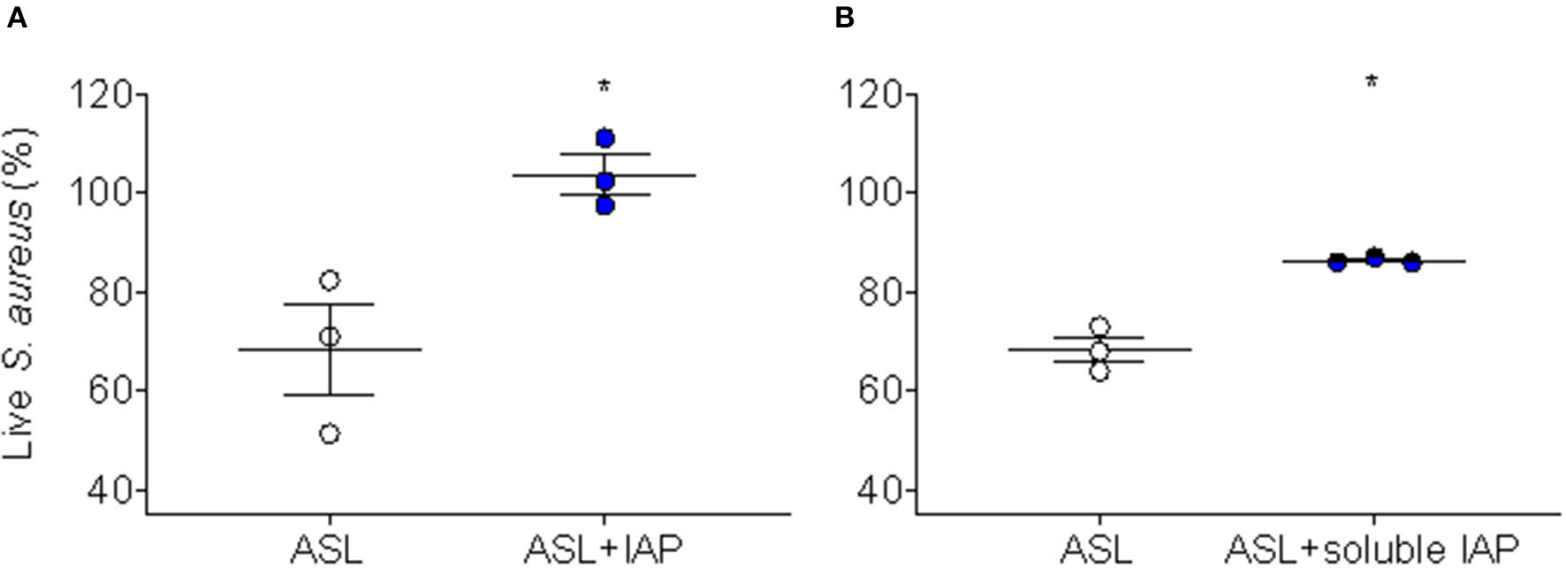
Inhibition of ASL antimicrobial activity using NIST IAP. **(A)** Percent of live *S. aureus* remaining within 10 min in the presence of ASL with and without exposure to whole-particle NIST IAP (50 μg/mL dose), compared to percent live bacteria in vehicle (10 mM NaPO_4_ buffer) with particle control at the same time-point; the whole IAP significantly inhibited large HAE cell ASL bacterial killing (*p* < 0.05), *n* = 3 from three different donors, ASL control was compared to ASL + IAP with a paired two-tailed *t*-test and error bars represent means and standard error of the means. **(B)** Live bacteria within 10 min compared to *t* = 0 min for ASL alone, and live bacteria within 10 min in the presence of the soluble portion of NIST IAP (50 μg/mL dose_initial_) was compared to *t* = 0 min at the same condition; the soluble portion of IAP significantly inhibited large HAE cell ASL bacterial killing (*p* < 0.05), *n* = 3 from three different donors. Error bars represent means and standard error of the means. Paired, two-tailed *t*-tests were used to compare the percent of live bacteria within 10 min to live bacteria at the initial reading (0 min), as the soluble portion did not have physical particles to interfere with RLUs, ^*^p < 0.05.

Because we have demonstrated CFA can inhibit ASL antimicrobial activity in human AECs (17), we were interested whether IAP would recapitulate this effect. To test this, we treated human AEC ASL with NIST IAP (dose = 50 μg/mL) for 45 min and then injected *S. aureus* and calculated immediate bacterial killing. The percentage of live *S. aureus* bacteria remaining after 2 min was compared to vehicle with particle control per condition (*n* = 3), Figure 2A.

We have reported that the soluble component of CFA does not inhibit human ASL antimicrobial activity (17); therefore, we decided to test whether the soluble portion of NIST IAP would inhibit human ASL antimicrobial activity. We therefore tested whether the soluble portion of the particle mixture (NIST IAP) may also inhibit bacterial killing using ASL from large human AECs. To test the effect of the soluble portion of IAP (whole-particle dose = 50 μg/mL), we treated human ASL with the supernatant of the particle mixture, then calculated the percentage of live *S. aureus* remaining within 10 min to the bacteria RLUs at *t* = 0 per condition, Figure 2B.

ASL in the presence of the whole-particle mixture inhibited ASL antimicrobial activity, Figure 2A (17). The soluble portion of NIST IAP also inhibited ASL immediate *S. aureus* bacterial killing (*p* < 0.05) (Figure 2B). However, the soluble portion of NIST IAP inhibited ASL antimicrobial activity less than the whole-particle mixture, Figures 2A,B.

### Study Population

We next assessed IAP from Iowa homes of subjects with and without active respiratory exacerbations. Subjects participating in our study who had experienced respiratory exacerbations over a 3-year period (“exacerbator group”), (*n* = 10) experienced a total of 64 exacerbations in the previous 3 years (mean = 2.1 exacerbations/year). Subsequent analyses were carried out to test for differences in the samples between the two groups. Table 1 describes study participant demographics.

**Table 1:** Study demographics.

**Demographics**	**Exacerbators (SD)**	**Non-exacerbators (SD)**	***P* value**
Age (y/o)	70 (8.1)	67 (6.6)	ns
Female	6	7	ns
GOLD status 1-2	5	2	ns
Exacerbations per year^**^	2	0	***<0.01***
Current smoker	1	1	ns
*Pack years*	41 (19.7)	28 (9.2)	0.07

*Number of study participants within exacerbator and non-exacerbator groups by demographics (n = 21); a GOLD status one or greater indicates COPD stage. Significance was assessed using unpaired t-tests (exacerbators vs. non-exacerbators). Bold font indicates significance, italicized font indicates a trend (p < 0.15), and significance was defined as p ≤ 0.05*,

** *p < 0.01*.

As seen in Table 2, the group without respiratory exacerbations had significantly more pet ownership, specifically cats (*p* ≤ 0.05). Additionally, the *p*-value for aluminum pan use was 0.11, with exacerbators reporting use of aluminum pans more than non-exacerbators.

**Table 2:** Survey responses regarding potential IAP generating sources.

**Survey response^**†**^**	**Exacerbators (SD)**	**Non-exacerbators (SD)**	***P*-value**
Time cleaning/week (h)	3.5 (3.2)	3.6 (2.6)	ns
Vacuum	9	9	ns
Dust	8	7	ns
Sweep	4	5	ns
Other cleaning	2	4	ns
Time cooking/week (h)	10 (5.9)	8.1 (4.7)	ns
Cook with gas	4	4	ns
*Cook using aluminum pan^‡^*	*4*	*1*	0.11
Cook using non-stick pan	6	4	ns
Cook using cast-iron	3	4	ns
Burn candles	3	2	ns
House heated by gas (inc gas fireplace)	8	10	ns
Garage attached to house	6	7	ns
Has pets^*^	***3***	***8***	***≤0.05***
Owns dogs	3	3	ns
Owns cats^*^	***0***	***4***	***≤0.05***
Total pets in home (#)^*^	***4***	***22***	***≤0.05***

† *Always indicates number of subjects in each category, unless otherwise noted*.

‡ *Some pans counted in both categories, i.e., metal and non-stick, if information available*.

Study-participant survey reponses (n = 21) related to life-style and housing conditions, significance was defined as p ≤ 0.05. Significance was assessed using unpaired t-tests (exacerbators vs. non-exacerbators). Bold font indicates significance (^*^), defined as p ≤ 0.05 and italicized font indicates a trend (p < 0.15).

We tested whether any bacteria could be found in the soluble portion of the filter samples from each home, and whether there was a difference between exacerbators vs. non exacerbators (Supplemental Table 2). Upon matrix-assisted laser desorption/ionization time of flight mass spectrometry analysis, ten IAP samples (48%) produced bacterial growth (Supplemental Table 2). Pathogens identified from the samples include: *Paenibacillus rhizosphaerae, Bacillus pumilus, Bacillus subtilis, Kocuria carniphila, Kocuria marina, Kocuria palustris, Micrococcus luteus, Micrococcus flavus, Microbacterium testaceum, Staphylococcus saprophyticus, Staphylococcus pettenkoferi, Pantoea calida, Pseudomonas fulva*, and 18 unknown bacterial species (not shown). Colony forming units ranged from 20–600,000 CFUs/mL. In addition, no significant difference in total bacterial CFUs was found between exacerbation groups (*p* = 0.22).

### Participant IAPs' Effect on Bacterial Growth, Biofilm Formation

Since NIST IAP enhanced bacterial growth and biofilm formation, we assessed whether IAP from Iowa homes would also consistently enhance *S. aureus* bacterial growth and *P. aeruginosa* biofilm formation. To test bacterial growth, we added IAP from each home to *S. aureus* and measured percent growth at 4 h, compared to the field blank control. To test biofilm formation, we exposed inoculator lids to challenge conditions containing each indoor PM sample for 24 h, then read OD_550_ compared to control.

**Figure 3:**
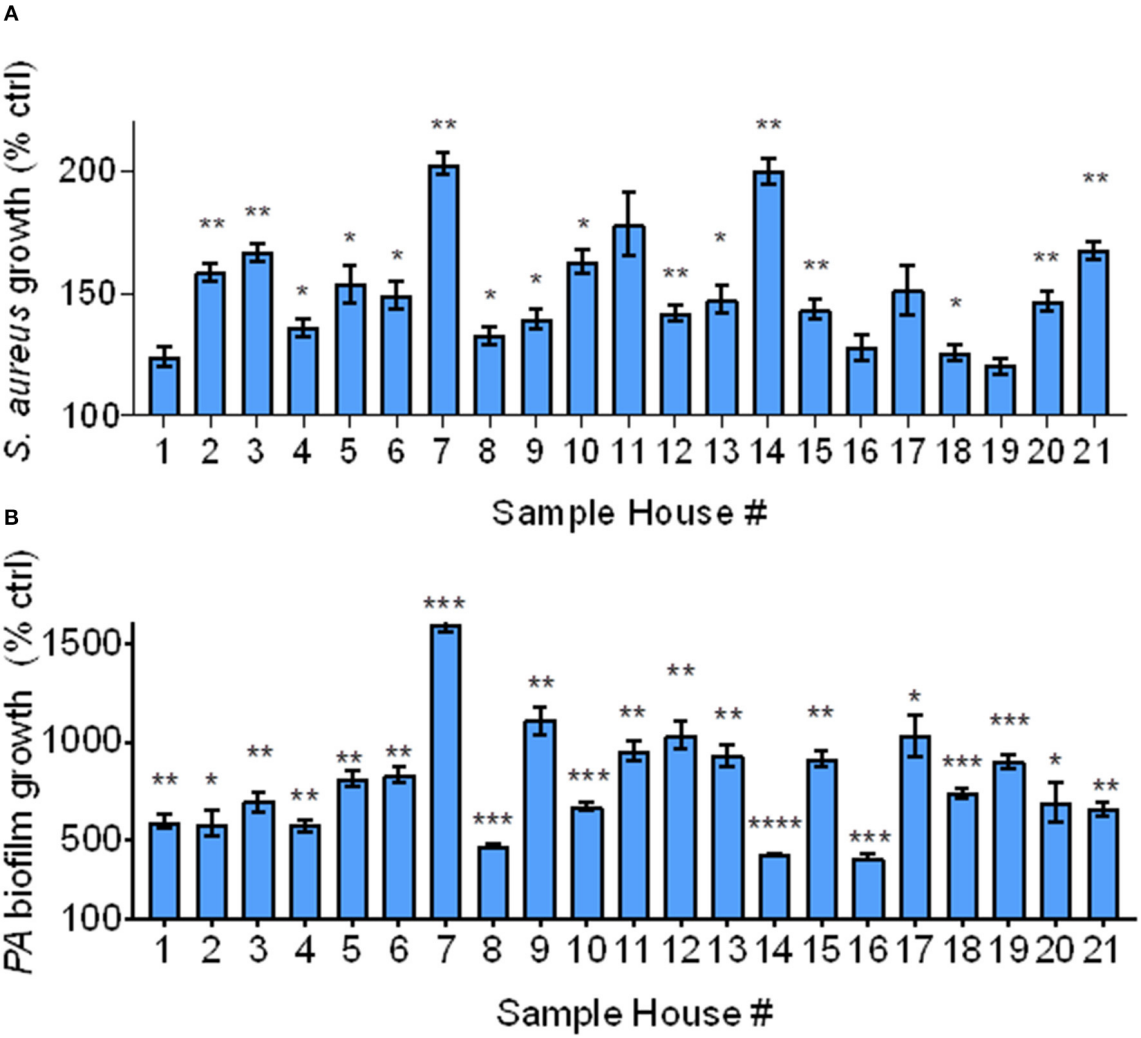
Bacterial growth and biofilm formation using Iowa house IAP. **(A)** *S. aureus* bacterial growth relative to the FB control at 4 h in the presence of soluble IAP from all homes, error bars represent means and standard error of the means and differences between FB control were compared to each condition using Brown-Forsythe and Welch ANOVA tests ^*^indicates *p* < 0.05, ^**^p < 0.01. **(B)** *P. aeruginosa* biofilm formation was higher in the presence of all IAP, error bars represent means and standard error of the means and differences between control compared to each condition were assessed using Brown-Forsythe and Welch ANOVA tests ^*^p < 0.05, ^**^p < 0.01, ^***^p < 0.001, ^****^p < 0.0001.

IAP from all houses enhanced bacterial growth after 4 h compared to the FB control. As shown in Figure 3A, bacterial growth significantly increased in all samples, except for in homes 1, 11, 16, 17, and 19. In homes whose IAP did not significantly promote bacterial growth, *p* ≤ 0.18.

As shown in Figure 3B, biofilm growth was greater than that of the FB control in all samples. The FB did not result in significant biofilm formation (data not shown), however all indoor samples demonstrated statistically significant biofilm formation (*p* < 0.05). We confirmed these findings using stereoscopic images (data not shown). We also looked at differences in bacterial growth and biofilm formation based on exacerbator status, but there was no difference (*p* = 0.78 and 0.93, for bacterial growth and biofilm formation, respectively), Supplemental Figures 1A,B.

### ASL Antimicrobial Activity

**Figure 4:**
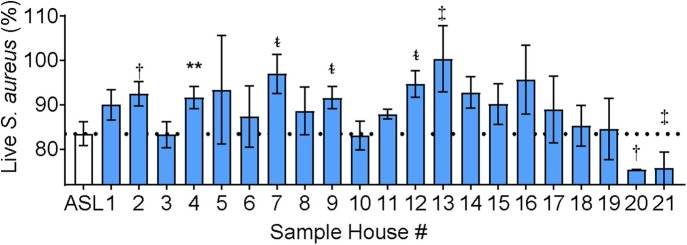
ASL antimicrobial activity in presence of Iowa house IAP. Percent live bacteria after treatment with ASL control (dotted line), and percent live bacteria after ASL was treated with indoor samples from all study participants. Error bars represent means and standard error of the means. IAP from house #4 significantly inhibited *S. aureus* bacterial killing, while IAP from five houses resulted in a trend of inhibited *S. aureus* killing, and two houses had a trend of enhanced *S. aureus* killing, (*p* ≤ 0.18). To test for differences between each house's IAP and ASL with the field-blank (filter) control, percent live bacteria after treatment with ASL control was compared using paired ᵵ-tests to the percentage of live bacteria remaining after ASL was treated with each participants' IAP, ^**^p < 0.01, ^†^*p* = 0.06–0.10, ^‡^*p* = 0.11–0.15, and ^t~^*p* = 0.16–0.20.

IAP differed from CFA in that the soluble component of NIST IAP inhibited ASL antimicrobial activity, unlike CFA (17). We next tested the effect of the soluble portion of IAP from houses of Iowa smokers on ASL antimicrobial activity. Figure 4 shows percent live bacteria of the ASL control, and percent live bacteria after ASL was treated with each participants' IAP samples. We report *p* < 0.20.

IAP from one home significantly inhibited ASL antimicrobial activity, and there was a trend (*p* ≤ 0.18) of inhibited ASL antimicrobial activity in five other homes (#2, 7, 9, 12, 13). In addition, there was a trend of enhanced ASL antimicrobial activity in two homes (#20 and 21).

**Figure 5:**
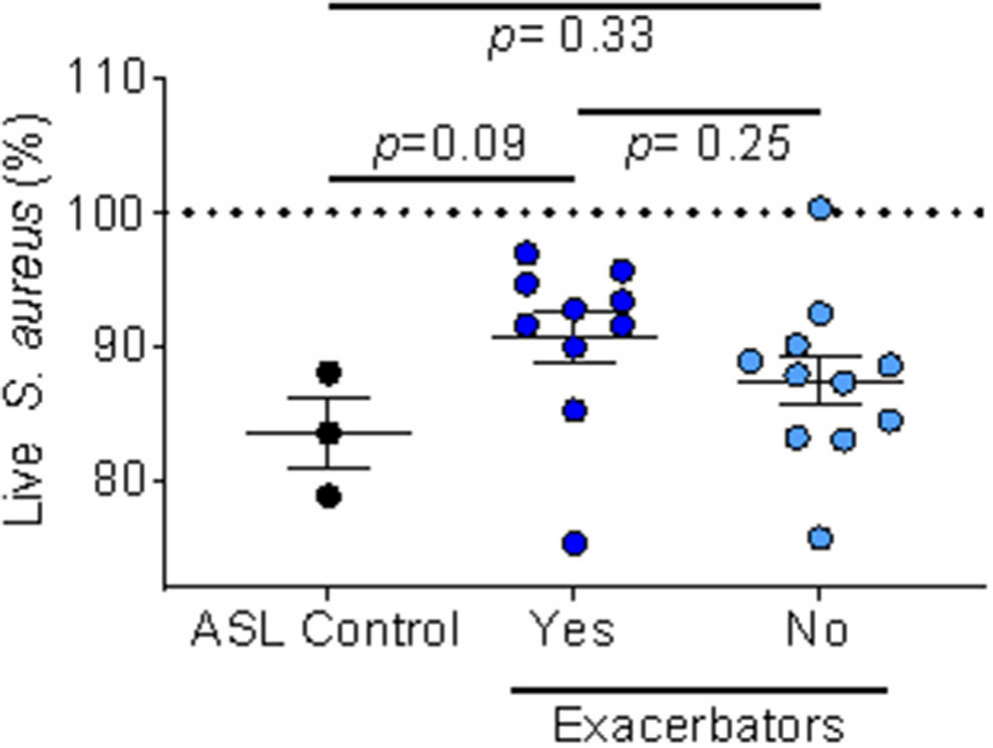
IAP's effect on ASL antimicrobial activity based on exacerbation history. Percent of live *S. aureus* at 16 min in the presence of IAP from exacerbators vs. non-exacerbators—values greater than the ASL control indicate inhibition of bacterial killing, while values lower than ASL alone indicate increased bacterial killing. Error bars represent means and standard error of the means. To test for differences between ASL with the field-blank control and the respiratory exacerbator group (and non-exacerbator group) we used unpaired *t*-tests.

We also looked at ASL antimicrobial activity using IAP from homes of respiratory exacerbators compared to bacterial killing in the presence of IAP of non-exacerbators, Figure 5.

We found a trend (*p* = 0.09) of inhibited ASL antimicrobial activity in the exacerbator group, compared to non-exacerbators, Figure 5. Additionally, IAP from subjects with a mild GOLD status appeared to greater influence ASL antimicrobial activity than IAP from subjects with a higher GOLD status (low GOLD status OR 1.32 [95% CI 0.93–1.88]; *p* = 0.12, High GOLD status OR 0.95 [95% CI 0.45–1.16]; *p* = 0.18).

### ASL Antimicrobial Activity in the Presence of Metals

The two most abundant metals (by mass fraction) in the Iowa samples were aluminum and magnesium (30). While we don't know the dose of Al or Mg in the soluble component of the NIST IAP, our 50 μg/mL dose contained 1.16 μg/mL Al, and 0.80 μg/mL Mg.

Divalent cations such as magnesium, have been reported to inhibit ASL antimicrobial activity (13). Copper is also a divalent cation, suggesting it may inhibit ASL bacterial killing; however, it is also a known antimicrobial agent, making it a metal of interest, especially given that IAP from the two houses had a trend of enhanced killing (#20 and 21). These homes had both the lowest amount of copper of any of the houses (#20), and the second highest amount of copper (#21), so we were therefore interested in copper's ability to kill bacteria.

**Figure 6:**
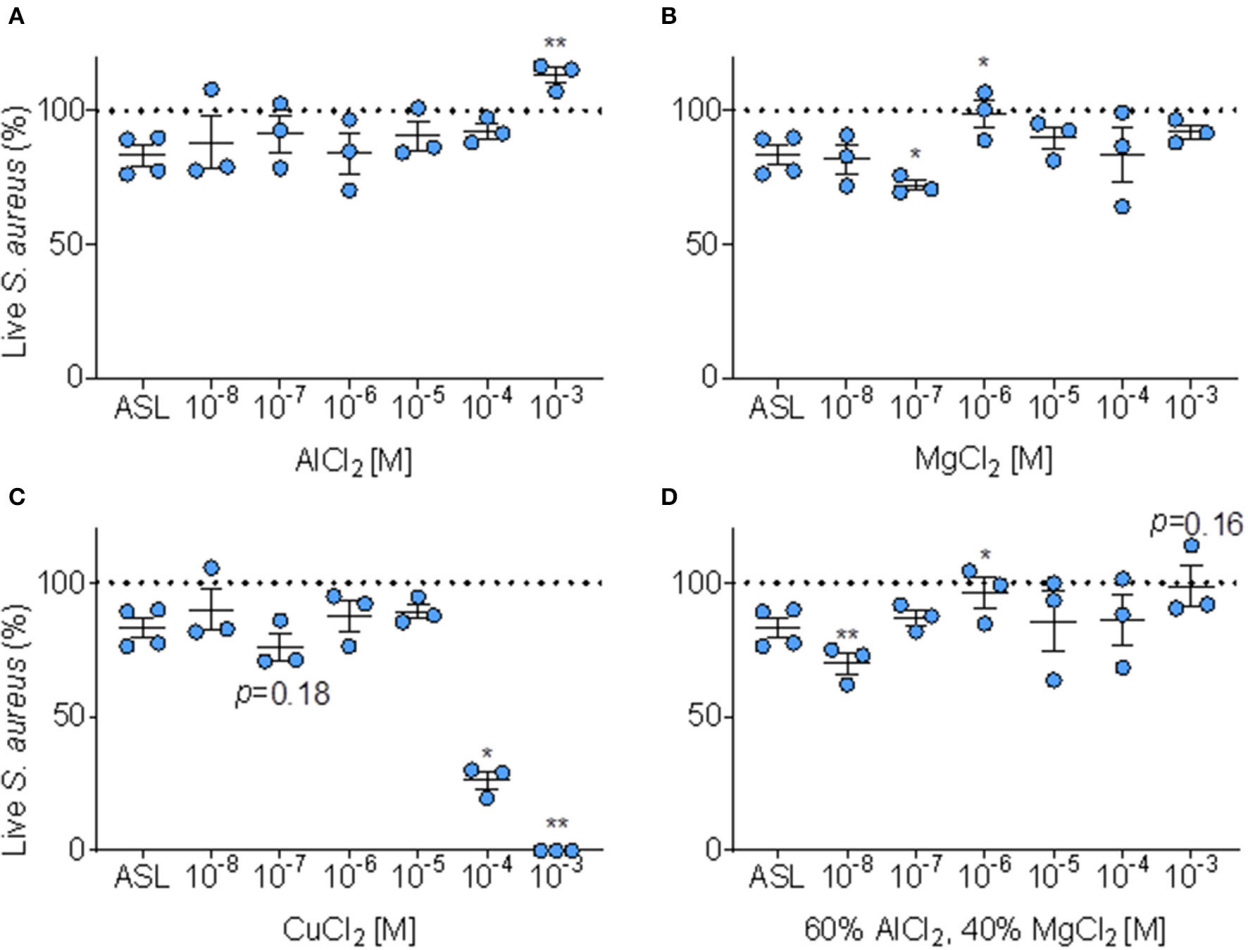
Effect of metals on ASL antimicrobial activity. **(A)** Percent of *S. aureus* bacteria alive immediately (≤ 10 min.) after exposure to ASL combined with six doses of AlCl_2_, error bars represent means and standard error of the means. **(B)** Percent *S. aureus* bacteria alive immediately (≤ 10 min.) after exposure to ASL combined with six doses of MgCl_2_, error bars represent means and standard error of the means. **(C)** Percent of *S. aureus* alive immediately (≤ 10 min.) after exposure to six doses of CuCl_2_ (proportions derived from sample means) relative to untreated ASL, error bars represent means and standard error of the means. **(D)** Percent *S. aureus* bacteria alive immediately (≤ 10 min.) after exposure to ASL combined with six doses of MgCl_2_ combined with AlCl_2_ in their average Iowa IAP proportions, error bars represent means and standard error of the means. *P*-values are generated from paired *t*-tests of percent live bacteria of each metal dose + ASL (at the same timepoint for each metal, ≤ 10 min) compared to ASL control at the same timepoint. **(A–D)** ^*^p ≤ 0.05 and ^**^p < 0.01.

We tested if adding Al^3+^, Mg^2+^, and Cu at six doses to the ASL altered immediate antimicrobial activity, compared to baseline ASL antimicrobial activity (Figures 6A–C).

As shown in Figure 6A, AlCl_2_ appeared to inhibit ASL antimicrobial activity in a dose-response manner; however, AlCl_2_ only significantly (*p* < 0.01) inhibited ASL antimicrobial activity at 1 mM. MgCl_2_ did not consistently affect ASL antimicrobial activity—it aided bacterial killing at 100 nM; however, inhibited ASL antimicrobial activity at 1 μM (*p* < 0.05) (Figure 6B). CuCl_2_ consistently had an antimicrobial effect on *S. aureus*, Figure 6C. At 100 nM, CuCl_2_ had a trend of enhanced killing (*p* ≤ 0.18), and at higher doses (≥100 μM), CuCl_2_ significantly enhanced bacterial killing, Figure 6C.

However, in our Iowa samples, we found that IAP from different homes had variable effects. Since Al and Mg are the most abundant metals (30), and are combined in the Iowa IAP samples, we decided to test whether a combination of these metals at similar proportions (60/40 of AlCl_2_ and MgCl_2_, respectively) would have different effects. When AlCl_2_ and MgCl_2_ are combined to the average proportion present in Iowa house IAP, the dose-response profile is dissimilar to either individual metal (enhanced killing at 10 nM and inhibition of killing at 1 μM), Figure 6D. There was no clear dose-response pattern influencing ASL antimicrobial activity for any specific metal analyzed, indicating the difficulty in predicting toxicity Figures 6A–D.

To test whether individual metal content explains the differences in modulation of ASL antimicrobial activity by IAP, we compared the weighted mass-fraction of each metal (30) to bacterial killing using a simple linear regression analysis. The mass fraction of Al, Mg, and Cu present in the IAP samples was not statistically significantly associated with the enhancement, nor inhibition, of bacterial killing (*R*^2^ = −0.00, 0.03, and 0.00, respectively). Therefore, the metal content of the particles was not enough to predict its biological effect on ASL antimicrobial activity.

## Discussion

The indoor environment can be a key source of pollution exposure. Indoor air pollution is linked to a multitude of negative human health outcomes (31, 32). We found that NIST IAP increases bacterial growth (Figure 1A) and biofilm formation (Figure 1B) in a dose response manner.

Prior work has implicated the insoluble portion of coarse PM as inducing toxic, proinflammatory responses to lung tissue (33); and our laboratory has previously demonstrated that the physical particle of coal fly ash adsorbs cationic antimicrobial peptides, reducing the bioavailability of these proteins to bind to bacteria, while the soluble component did not inhibit human large ASL antimicrobial activity; however, research in this field is limited (17, 34). We were therefore interested in assessing whether the soluble portion of the IAP mixture may also inhibit ASL antimicrobial activity. Not only did the whole-particle mixture of IAP inhibit ASL antimicrobial activity (Figure 2A), but the soluble portion of the particle mixture also significantly inhibited ASL antimicrobial activity (Figure 2B), suggesting the involvement of other mechanisms of ASL antimicrobial activity impairment.

COPD subjects with moderate to severe COPD have previously been shown to experience increased respiratory symptoms, medication use, and risk of severe exacerbations alongside increased PM_2.5_ levels in the primary household living area (35). Also, it was recently reported that symptomatic current and former smokers with preserved lung function have respiratory exacerbations (36). We were therefore interested in determining whether IAP from houses of smokers with and without a history of respiratory exacerbations would differentially affect bacterial growth (*S. aureus*) biofilm formation (*P. aeruginosa*), and ASL antimicrobial activity. No differences were found between participants experiencing respiratory exacerbations and those without—IAP promoted bacterial growth and biofilm formation independent of exacerbation status (Figures 3A,B). Bacterial growth was variable but consistent in all cases. Enhanced bacterial growth in the presence of IAP implies that, upon exposure to pathogens, the potential for bacteria to remain viable in subjects who have inspired IAP may be greater.

While we found no differential effect of IAP on bacterial growth and biofilm formation between groups (those experiencing recent respiratory exacerbation and those without respiratory exacerbation), there was a differential effect of ASL antimicrobial activity. Iowa house IAP samples either impaired, had no effect, or had a trend of enhanced ASL antimicrobial activity. We also found a trend indicating that home IAP of respiratory exacerbators may preferentially inhibit ASL antimicrobial activity compared to IAP from homes of non-exacerbators (Figure 5). The *p*-value may be explained by the low power of the sample size. It is known that host innate immunity is compromised in COPD subjects due to a number of factors including impaired mucociliary clearance and reduced AMPs, among others (37). IAP from houses of exacerbators may preferentially attenuate the airway's natural defense system, potentially playing an important role in innate immunity. Additionally, there appeared to be a more pronounced effect of IAP on the mild GOLD status subjects. While not statistically significant, it suggests a differential effect of air pollution depending on underlying COPD severity. We speculate that this result reflects that the home environment of smokers (ex and current) *without* established COPD (GOLD 0) may impair airway antimicrobial activity more so than the home environment of those with established COPD (GOLD ≥ 1), thus contributing to an increased risk of developing respiratory exacerbations.

Our cohort of subjects was relatively homogenous in terms of age, gender, smoking status, geographical location, and typical time spent performing house-hold activities. However, two distinct differences emerged between the non-exacerbator and exacerbator groups. Subjects without a recent history of respiratory exacerbation more frequently reported owning pets, and had significantly more pets (cats), than those with recent respiratory exacerbation. This finding may reflect health status, e.g., those not currently experiencing exacerbations are more capable of handling the demands of a domestic animal but could also be indicative of previous pet-ownership. The link between cat ownership and sensitization (allergy, asthma) is uncertain, but dog ownership has been shown to either have no effect, or a positive effect on reducing sensitization (38). More research on respiratory exacerbations and household pet ownership is warranted. Additionally, respiratory exacerbators reported cooking with aluminum pots/pans, Table 2, while only one participant in the non-exacerbator group noted using aluminum. Although not statistically significant, the difference is noteworthy considering our findings regarding the inhibitory effect of aluminum on airway innate immunity.

Non-exacerbator samples had twice as many micro-organism species identified in filters from their homes compared to the exacerbator group, Supplemental Table 2. This finding is not surprising considering non-exacerbators owned more animals. When comparing total CFUs based on exacerbation status, there was no significant difference between the two groups. *Pseudomonas* and *Staphylococcus species* (2) were found in IAP samples (House #13 and 18). Both are important pathogens in the progression of lung disease. *Staphylococcus aureus* and *Pseudomonas aeruginosa* are common pathogens (gram positive and negative) found in immunocompromised patients (39–41). In addition, *P. aeruginosa* growth is enhanced in the presence of ambient air pollution, and *S. aureus* bacterial growth is enhanced in the bronchoalveolar lavage of smokers (15, 16).

Antimicrobial peptide activity in the ASL is known to be disrupted by certain divalent cations in the mM range (13, 42). Divalent cations are thought to bind to negatively charged bacteria, rendering bacteria less capable of binding to cationic antimicrobial peptides present in the ASL (17). Interestingly, only IAP from COPD house number 4 statistically inhibited bacterial killing (Figure 4). Although some IAP appeared to have a trend of enhanced ASL antimicrobial activity, this is not necessarily indicative of a positive health outcome, as PM_2.5_ can be pro-inflammatory (induce cytokine release) and cytotoxic, which is in part mediated by transition metals (43, 44).

Previous work has shown in-home endotoxin exposures are not the causative agent in worsening of COPD symptoms and, because iron can alter bacterial growth, we hypothesized that metals may play an important role in respiratory exacerbations (16, 45). However, no statistically significant association between specific metals (mass fraction nor soluble component) in the samples, such as iron, and bacterial growth was found. This may be due to the small sample size, or the wide variety of metals present in the samples and distinct combinations of metals potentially affecting bacteria in unique ways.

We analyzed Al^3+^ and Mg^2+^ in a dose-response manner to test their effect on bacterial killing and found that at 1 mM (AlCl_2_), and 1 μM (MgCl_2_), these metals inhibited antimicrobial activity, Figures 6A,B. However, at 100 nM MgCl_2_, ASL bacterial killing was enhanced, Figure 6B. Polyvalent cations, Al^3+^ and Mg^2+^ have a complex effect on ASL bacterial killing when combined (Figure 6D). Aluminum, a polyvalent cation, seems to have a trend of inhibiting in a dose-response manner. When combined to their average indoor PM proportion, Al^3+^ and Mg^2+^ resulted in enhanced killing at a low dose (10 nM), yet there was more inhibition at higher doses (1 μM and 1 mM). This presents a novel dose-response profile compared to the individual metals, and confirms the metals behave distinctly when combined.

Interestingly, Copper, which is commonly found in building furnishings and generated during cooking (46) has been shown to be an independent antimicrobial agent (47–49) and, as expected, at doses >10 μM it was sufficient to kill bacteria independent of ASL activity (Figure 6C). The half maximal inhibitory concentration 50% (IC_50_) of ASL with CuCl_2_ occurred at ~194 μM (not shown), while CuCl_2_ combined with water also immediately killed *S. aureus* independently of ASL antimicrobial activity (Supplemental Figure 2). However, bacterial killing occurred at lower doses of copper combined with ASL (IC_50_ ~ 58), compared to copper alone (IC50 ~ 194 μM), suggesting the antimicrobial effect of copper appears to be synergistic with ASL compared to water alone.

The volume of ASL used in our assay was 10 μL, healthy human lung has an ASL height of ~40 μm (50) given the surface of a human lung is ~70 m^2^ (51), this would translate to an approximate ASL volume of 28 ^*^ 10^−4^ m^3^. Soluble metal concentrations in the house samples for Al^3+^, Cu^2+^, and Mg^2+^ in our study ranged from 0 to 29 μg/mL. These appear to be biologically relevant doses, as reports of lung tissue loads (analyzed from deceased males) from two continents (*n* = 36), were 43–67 mg/kg, and rat serum Al^3+^ levels were ~21 μg/L (52). Magnesium levels remain fairly constant across all species, as all mammalian cells contain free cytosolic Mg^2+^ from 0.25 to 1 mM (53).

One of the limitations of our study is that all our experimental media had a pH of 7.4–7.54 and had identical ionic concentration at baseline. Therefore, our results are evaluating only antimicrobial peptides and proteins in the ASL, and not assessing other variables present in the ASL that could potentially affect antimicrobial activity. Additionally, our study is a pilot study, consisting of *in vitro* data and our sample size is a limitation in determining statistical significance.

Our study confirmed that NIST standard PM from the indoor environment affected mechanisms demonstrably culpable in the development of airway infection, including enhanced bacterial growth, biofilm formation and impaired ASL antimicrobial activity. Additionally, these results were translated in a high-risk study population (smokers) and we demonstrated that two of the same mechanisms (bacterial growth and biofilm formation) were recapitulated using IAP from homes of smokers, and additionally, ASL antimicrobial activity may be preferably compromised using the IAP from homes of subjects with history of frequent respiratory exacerbations. This finding was supported by the result that subjects with a mild GOLD status had a much higher OR of impairing innate immunity than those with a high GOLD status. We consider these mechanistic insights in a translational setting our study strengths.

## Conclusion

We demonstrated that NIST IAP and Iowa house IAP enhanced bacterial growth and biofilm formation. NIST IAP significantly impaired ASL antimicrobial activity, while we found a trend of impaired ASL antimicrobial activity using Iowa house IAP from homes of subjects with a history of frequent respiratory exacerbations. We found that although specific metals may help explain mechanisms of impaired airway innate immunity, the effect of combined metals on airway innate immunity remains to be explored.

## Data Availability Statement

The datasets generated for this study are available on request to the corresponding author.

## Ethics Statement

The studies involving human participants were reviewed and approved by University of Iowa IRB. The patients/participants provided their written informed consent to participate in this study.

## Author Contributions

ESta, RM, JN, GP, ESto, OC, TH, and RB contributed to the data acquisition and analysis. ESta, TP, ESto, PP, JZ, and AC contributed to the concept and design of the work. ESta and AC drafted. XL, ESta, AC, and JZ revised the manuscript. All authors agree to be accountable for the work.

### Conflict of Interest

The authors declare that the research was conducted in the absence of any commercial or financial relationships that could be construed as a potential conflict of interest.
