# Supplementary material for: Indoor Particulate Matter From Smoker Homes Induces Bacterial Growth, Biofilm Formation, and Impairs Airway Antimicrobial Activity. A Pilot Study
Source: Front Public Health. 2020 Jan 24;7:418. doi: 10.3389/fpubh.2019.00418 (PMC6992572; doi:10.3389/fpubh.2019.00418)
Supplement: Supplementary file 1 [file Data_Sheet_1.docx]

**SUPPLEMENTAL METHODS**

*Bacterial culture*We used a bioluminescent strain of *S. aureus* (Xen29, Caliper LifeSciences Bioware™), as previously described (Buonfiglio et al. 2017). Briefly, bacteria were plated from a glycerol stock onto tryptic soy agar plates (with 60 µg/mL kanamycin, to maintain bioluminescent selection) and grown in an incubator overnight at 37°C. Four bacterial colonies were selected, suspended in tryptic soy broth (TSB, BD Difco), and cultured overnight with kanamycin in a rotating (260 rpm) 37°C incubator. In the morning, we diluted the bacteria (1:50) in identical media, and upon log-phase growth, we centrifuged (16 x g, 2 min), washed (PBS -/-) (Gibco® Life Technologies), and resuspended bacteria in a 10 mM NaPO_4_ buffer containing TSB (concentration_Final_ = 1%). Bacteria were diluted to optical density (OD) 600 nm, conc_Final_ = 0.01 (~3x10^6^ CFUs/mL). Bacterial luminescence was read at 527 nm and bacteria were quantified by relative light units (RLUs), which we confirmed with CFUs (Buonfiglio et al. 2017).

**RESULTS**

**Supplemental Table 1.** Survey questions completed by study participants.

| How many hours a week is your home cleaned? |
| --- |
| What is the primary method of cleaning (dusting, vacuuming, sweeping, etc.)? |
| How many hours a week do you cook? |
| What kind of materials do you cook with? Are your pots, pans, and cooking utensils primarily metal, wood, plastic, etc.? |
| How many days a week is tobacco smoked in the house? |
| Do you know how many cigarettes are smoked in your home a day? |
| How many days a week are candles burned in the house? |
| Is your home heated by fireplace, gas, electric, central air or radiators? |
| Do you have a garage attached to your home? |
| How many days a week do you keep the windows open? |
| How many hours a day do you normally keep them open? |
| Are the walls painted with lead based paint? |
| How many pets live in the house? |
| What kind of pets are they? |
| Has there been recent construction or renovation inside the house? |
| What is the rough square footage of the home? |

**Supplemental Table 2.** **Species of bacteria found in Iowa home filter samples.** Bacteria and colony forming units (per mL) identified in IAP suspensions from homes with identifiable bacterial growth. Exacerbator homes identified by bold type-face.

| **Home (#)** | **Bacteria ID** | **CFU/mL** |
| --- | --- | --- |
| **1** | ***Paenibacillus rhizosphaerae*** | **60** |
| **1** | ***Bacillus pumilus*** | **200** |
| 9 | *Kocuria marina* | 4000 |
| 11 | *Micrococcus luteus* | 360 |
| 11 | *Microbacterium testaceum* | 20 |
| 11 | *Kocuria marina* | 140 |
| 11 | *Bacillus subtilis* | 20 |
| 11 | *Kocuria palustris* | 400 |
| 13 | *Kocuria marina* | 60 |
| 13 | *Staphylococcus saprophyticus* | 80 |
| 13 | *Micrococcus luteus* | 60 |
| 13 | *Staphylococcus pettenkoferi* | 20 |
| 13 | *Kocuria palustris* | 60 |
| 15 | *Pantoea calida* | 3600 |
| 17 | *Micrococcus luteus* | 60 |
| 17 | *Micrococcus flavus* | 80 |
| 17 | *Kocuria carniphila* | 20 |
| **18** | ***Pseudomonas fulva*** | **600000** |

**Supplemental Figure 1A.** Percent *S. aureus* growth compared to the field-blank control after four hours in the exacerbator group (n=10) relative to the non-exacerbators (n=11). Exacerbation status of “yes” indicates the study participant experienced active respiratory exacerbations (at least two) over the previous three years while exacerbation status of “no” indicates the study participant experienced zero respiratory exacerbations over the previous three years **B.** *P. aeruginosa* biofilm formation after 24-hours in the presence of 10 µL of IAP samples (percent growth relative to control) compared between the two groups

**Supplemental Figure 2.** CuCl_2_ is antimicrobial independently of ASL, *S. aureus* bacterial challenge in the presence of four doses of CuCl_2_ suspended in water.
